# Measuring lung mechanics in patients with COPD using the handheld portable rapid expiratory occlusion monitor (REOM): A cross‐sectional study

**DOI:** 10.14814/phy2.70307

**Published:** 2025-04-07

**Authors:** Felix‐Antoine Coutu, Dany Malaeb, Olivia C. Iorio, Seyedfakhreddin Nabavi, Tom McFarland, Lennart K. A. Lundblad, Ron J. Dandurand, Stewart B. Gottfried, Bryan A. Ross

**Affiliations:** ^1^ Respiratory Epidemiology and Clinical Research Unit, Centre for Outcomes Research and Evaluation Research Institute of the McGill University Health Centre Montreal Quebec Canada; ^2^ Department of Medicine McGill University Montreal Quebec Canada; ^3^ Meakins‐Christie Laboratories, McGill University Montreal Quebec Canada; ^4^ THORASYS Thoracic Medical Systems Inc. Montreal Quebec Canada; ^5^ Montreal Chest Institute, McGill University Health Centre Montreal Quebec Canada; ^6^ Lakeshore General Hospital Pointe‐Claire Quebec Canada; ^7^ Ste‐Anne Hospital Ste‐Anne‐de‐Bellevue Quebec Canada; ^8^ Translation Research in Respiratory Diseases Program Research Institute of the McGill University Health Centre, McGill University Montreal Quebec Canada

**Keywords:** chronic obstructive pulmonary disease (COPD), handheld portable device, interrupter technique, oscillometry, respiratory resistance

## Abstract

While conventional spirometry is associated with strenuous “forced” maximal respiratory maneuvers and infection control implications, oscillometry is not associated with these issues. However, portability, convenience of use, and interpretation remain common limitations to both techniques. This study tested the concordance and agreement between resistance measurements obtained from the handheld portable REOM device (R_eo‐f_, R_eo‐s_) with those from conventional oscillometry (R_19_, R_5_) in PFT‐confirmed “mild” (GOLD 1) and “very severe” (GOLD 4) COPD. Unadjusted and adjusted concordance (Spearman correlation) and agreement (Bland–Altman tests) served as co‐primary outcomes. Discrimination between GOLD 1 and 4 COPD (Wilcoxon rank sum test, Support Vector Machine (SVM) classifier) and patient user experience (System Utility Scale (SUS), Participant Satisfaction Survey (PSS)) served as secondary outcomes. In 17 participants (GOLD 1 *n* = 9, GOLD 4 *n* = 8), adjusted R_5_‐R_eo‐s_ (0.95 [0.81, 0.98]) and R_19_‐R_eo‐f_ (0.93 [0.79, 0.99]) correlations were very strong, as was agreement (mean differences: −0.07, 0.08, respectively). Statistically significant between‐group differences were observed for all four resistance parameters. R_eo‐s_ in particular exhibited perfect discrimination between GOLD 1 and 4 disease, with some minor misclassification by R_eo‐f_, R_5_ (*n* = 1 each) and R_19_ (*n* = 4). User experience scores were excellent. These results support the capacity for REOM as a novel, complementary diagnostic device in COPD.

## 1 INTRODUCTION

Chronic obstructive pulmonary disease (COPD) is a highly prevalent chronic respiratory condition (Stolz et al., 2022) with escalating morbidity and mortality rates worldwide (Yan et al., 2017). COPD is the third‐leading cause of death globally and is characterized by chronic symptoms including breathlessness, wheeze, cough, and chest tightness as well as by gradual lung function decline (Global initiative for chronic obstructive lung disease, 2024). Patients with COPD experience frequent unscheduled health service use and prolonged hospital stays, which can substantially affect quality of life (Michalovic et al., 2020) and are associated with very high healthcare costs (Bakthavatsalu et al., 2023). The growing global burden of COPD and of COPD‐related admissions (Amegadzie et al., 2023; Boers et al., 2023) is placing increasing demands on healthcare systems, which often face limited capacity and resources (Hermus et al., 2012), leading to the investigation of alternate care models including home lung function monitoring.

The hallmark feature of COPD is the sustained reduction in expiratory airflow, commonly referred to as airflow obstruction, or limitation, for which spirometry is used as the prevailing “gold standard” for detection, diagnosis, and severity classification (Fry & Hyatt, 1960; Kouri et al., 2021; Osterling et al., 2014; Tiffeneau & Pinelli, 1947; Yentes et al., 2020). Spirometry is also used clinically in a serial/interval manner to monitor outpatient lung function and in order to help determine appropriate pharmacotherapy, evaluate response to therapy, and to inform overall prognosis (Bourbeau et al., 2023; Global initiative for chronic obstructive lung disease, 2024). Notably, however, the spirometry testing process requires substantial patient cooperation and coordination, as well as the capacity and willingness to participate in difficult and strenuous “forced” maximal respiratory maneuvers. The inability to do so can be a major factor in obtaining accurate and reliable results, which can interfere with the ability to establish a reliable diagnosis and can complicate long‐term management (Bates et al., 2011; King et al., 2020). The forced breathing maneuvers necessary can be particularly challenging for older adults (Incalzi et al., 2014; Melo et al., 2019; Pezzoli et al., 2003), which represents the majority of the COPD population, as well as for patients with advanced disease (Global initiative for chronic obstructive lung disease, 2024). The routine use of spirometry in the outpatient management of COPD also represents a burden for healthcare systems in terms of financial and personnel resources, and represents an infection control vulnerability as was highlighted during the COVID‐19 pandemic (Kouri et al., 2020; Lundblad & Chow, 2020). The operating costs (necessary equipment and skilled personnel) of on‐site spirometry interferes with uptake and regular use in community clinical practice, further exacerbating long waitlists and poor access to large central pulmonary function labs (Lundblad, Blouin, et al., 2021).

The rapid expiratory occlusion monitor (REOM) device is portable, lightweight and can be paired wirelessly to a smartphone or tablet (Lundblad, Blouin, et al., 2021). With an output of only two resistance values, it requires minimal training to collect and interpret the data. The two described values, referred to as “Reo – fast” (R_eo‐f_) and “Reo – slow” (R_eo‐s_), have been shown to correlate closely with spectral oscillometry‐obtained resistance parameters at high (19 Hz) and low (5 Hz) frequencies, respectively, in children with asthma (Lundblad, Blouin, et al., 2021). Given the inherent differences in lung mechanical properties, however, between the dynamic process of lung development throughout childhood versus at maturity (e.g., pediatric vs. adult populations) (Wheeler et al., 2011) as well as between different obstructive diseases (e.g., asthma versus COPD) (Rogliani et al., 2016), a population‐ and disease‐specific investigation is necessary to bridge the existing knowledge gap on the use of the REOM device for the management and monitoring of lung function in the large and growing adult COPD patient population.

The primary objective of the present study is to determine the concordance and agreement between the two parameters obtained from the REOM device, R_eo‐s_ and R_eo‐f_, and the conventional resistance parameters obtained by standard spectral oscillometry at 5 and 19 Hz (R_5_ and R_19_, respectively) in adults with mild (i.e., GOLD 1) or very severe (i.e., GOLD 4) COPD as confirmed by conventional lung function testing. The secondary objectives are to investigate the discriminative capacity to distinguish between mild and very severe COPD, and to determine the COPD patient user experience with the REOM device. We hypothesize that high concordance and agreement will be observed between R_5_ and R_eo‐s_, as well as between R_19_ and R_eo‐f_, in the adult patient population with mild and very severe COPD. We also hypothesize that the REOM resistance measurements, in particular R_eo‐s_, will be able to distinguish between “mild” and “very severe” COPD, and that participants will report a positive user experience with the REOM.

## 2 MATERIALS AND METHODS

### 2.1 Study design and eligibility criteria

This single‐site, cross‐sectional observational study included participants recruited from the subspecialized COPD clinical program of the Montreal Chest Institute (MCI) of the McGill University Health Centre (MUHC). The study was approved by the MUHC Research Ethics Board (REB study number: 2023‐9001). All participants provided both written and verbal informed consent prior to participation. This study received approval from Health Canada for Class II Investigational Testing Authorization (ITA: 355823) and was registered on ClinicalTrials.gov (NCT 05913323).

Participants were recruited between August 2023 and August 2024. Eligibility criteria were as follows: male or female patients aged ≥40 years, former or current smokers with a ≥10 pack‐year smoking history, diagnosis of either “mild” (GOLD 1) or “very severe” (GOLD 4) COPD as confirmed by spirometry (Global initiative for chronic obstructive lung disease, 2024), and the ability to provide informed consent. Participants were excluded from the study if they did not have a prior diagnosis of COPD, if they had comorbid asthma, if they required continuous supplemental home oxygen, if they had recently experienced an acute exacerbation of COPD (within 4 weeks of testing), if they had a chronic or suppurative respiratory infection, if they also had one or more absolute contraindications to standard pulmonary function testing (Graham et al., 2019), or if they were unable to participate due to any physical/cognitive barriers. The GOLD severity criteria for COPD classifies the disease based on the post‐bronchodilator forced expiratory volume in the first second (FEV_1_) as a percentage of predicted normal: GOLD 1 (mild) COPD corresponds to a forced expiratory volume in the first second (FEV_1_) ≥80% predicted, GOLD 2 (moderate) COPD corresponds to 50% ≤ FEV_1_ < 80% predicted, GOLD 3 (severe) COPD corresponds to 30% ≤ FEV_1_ < 50% predicted, and GOLD 4 (very severe) COPD corresponds to FEV_1_ < 30% predicted (Global initiative for chronic obstructive lung disease, 2024). The data of prescreened participants (based on pre‐existing spirometry results) who were initially recruited however ultimately did not demonstrate “mild” or “very severe” fixed airflow obstruction on the day of in‐person testing by full pulmonary function test (PFT) were excluded from data analysis.

### 2.2 Recruitment and protocol

All testing was performed at the Centre for Innovative Medicine (CIM) of the MUHC at a single in‐person visit. Standard lung function testing (including a full PFT) could not precede oscillometry and handheld expiratory occlusion testing (King et al., 2020) and was thus the last of the three tests performed in each recruited participant. Chart review was therefore conducted to pre‐screen for COPD diagnosis meeting criteria on spirometry and to determine likely airflow obstruction severity. Sex assigned at birth was collected from the electronic chart, and gender identity was collected from participant self‐report (Heidari et al., 2019).

Following recruitment and the collection of baseline demographic information, the first test performed at the in‐person visit was the determination of R_5_ and R_19_ with standard spectral oscillometry (tremoflo C‐100, Thorasys Thoracic Medical Systems Inc., Montreal, Canada) using the 5–37 Hz waveform. Between 3 and 6 standard oscillometry measurements were taken, in accordance with the European Respiratory Society (ERS) technical standards, until a coefficient of variance (CV) ≤10% was achieved for the resistance at the lowest frequency (King et al., 2020). The second test conducted was the determination of R_eo‐s_ and R_eo‐f_ using the REOM device (Spiro‐tech Medical Inc., Montreal, Canada). The REOM device was linked to a tablet via Bluetooth to display and store the data. The technical approach used during testing with the REOM device followed the standards recommended for spectral oscillometry (King et al., 2020), except that, notably, the device user manual does not mandate a cheek‐hold technique when collecting data, given the handheld portable design and the intention for potential autonomous home use. Three REOM tests per participant were taken, each consisting of a minimum of 5 and a maximum of 11 breathing cycles. A measurement was automatically considered valid by the device if at least three breaths were free of artifact or forceful expiration.

In an exploratory manner, to determine whether the REOM device could maintain acceptable accuracy while being used autonomously by participants without requiring additional in‐person assistance, the performance of the REOM device was assessed by collecting three sets of tests: (1) participants only held the REOM device without supporting their cheeks, as instructed in the user manual (no cheek hold); (2) participants held both their cheeks with one hand alone (thumb on one cheek, fingers on the other cheek) and used the other hand to hold the REOM device (one‐handed cheek hold); and finally (3) participants used both hands to hold their cheeks while a technician held the REOM device for the participant during testing (two‐handed cheek hold).

As the final test, each participant performed a full PFT which included pre‐ and post‐bronchodilator spirometry (to confirm fixed airflow obstruction and proper GOLD classification), plethysmography (lung volumes), and diffusion capacity testing (Graham et al., 2019). PFTs were performed using standard equipment (Medisoft BodyBox) and in accordance with the American Thoracic Society (ATS) criteria (Stanojevic et al., 2022). The order of tests at the in‐person visit were intentional since the forced maneuvers required by spirometry are known to alter lung mechanics (Fish et al., 1978; Pellegrino et al., 1996).

At the end of the in‐person study visit, to evaluate the experience with the REOM device specifically from the perspective of the COPD patient user, each participant completed two questionnaires. The first was the validated System Utility Scale (SUS), designed to assess the general usability of the device (Brooke, 1996). The SUS consists of 10 questions answered on a Likert scale and has been recognized as a practical, reliable, and versatile tool for evaluating the user experience with technology in previous studies (Iorio et al., 2024; Keogh et al., 2020; Lewis & Sauro, 2009; Liang et al., 2018). The SUS yields a single score ranging from 0 to 100, representing a composite measure of overall usability. The second questionnaire, the Participation Satisfaction Survey (PSS), consisted of a total of five questions either graded on a 5‐point Likert scale or in an open‐ended format (see Tables S1–S4). The PSS was previously used in determining the REOM user experience in asthma (Lundblad, Blouin, et al., 2021).

### 2.3 REOM device signal processing

As described in detail and illustrated previously in published work (Lundblad, Blouin, et al., 2021), the flow signal obtained from the REOM device during expiration over time was natural log‐transformed and used to measure resistance, the ratio of pressure and flow, at two distinct time intervals: (i) at the time of maximum flow achieved within 50 milliseconds (ms) immediately after shutter release (i.e., at the point of reversal of expiratory occlusion—“fast” signal); and (ii) the y‐intercept derived from the slope of the natural log‐transformed flow‐time curve between 5 and 200 ms after shutter release (i.e., “slow” signal). The pressure used for the determination of both resistance estimates was the peak pressure of 400 Pa measured during the occlusion stage, shortly preceding shutter valve opening. The two resultant measurements were labeled as R_eo‐f_ and R_eo‐s_, respectively (Lundblad, Blouin, et al., 2021).

### 2.4 Statistical analysis

A priori sample size was estimated by determining the number of paired observations needed to test for a significant correlation coefficient between REOM‐obtained and standard spectral oscillometry‐obtained parameters. To test whether the observed correlation differs from 0, with an assumed 45% coefficient of determination or greater between the REOM and spectral oscillometry in the adult COPD patient population (two‐tailed test with power 0.8 and alpha of 0.05) (Negida, 2020), *n* = 16 participants would be required in order to adequately test the primary outcome. Factoring in the possibility of attrition and/or possible data acquisition or quality issues, the recruitment of *n* = 19 participants was targeted.

Demographic information and lung function results were presented either as means with standard deviation (SD) or as medians with interquartile range (IQR), as appropriate. The co‐primary study outcomes were the correlation (using Spearman correlation testing) and the agreement (using Bland–Altman testing) between the resistance measurements obtained by the REOM device and those obtained by the standard spectral oscillometer. Analyses were adjusted for age, sex, smoking, body mass index (BMI), and disease severity. Discrimination was tested first with a Wilcoxon rank sum test and then with a general Support Vector Machine (SVM) (Pisner & Schnyer, 2020) classifier to determine the capacity of REOM‐obtained resistance values to accurately and precisely classify COPD severity as “mild” (GOLD 1) or “very severe” (GOLD 4). Patient user experience (SUS and PSS) results in all participants and by group were presented descriptively. Finally, the Friedman test was used to detect any statistically significant differences between the three sets of REOM device measurements obtained: no cheek hold, one‐handed cheek hold, and two‐handed cheek hold. Where a main effect was observed, a Bonferroni post hoc test was performed to locate differences between the three sets of tests. A *p* value of <0.05 was considered to be statistically significant. All statistical analyses were performed in MATLAB Software, version 2024.a (MathWorks, 2024).

## 3 RESULTS

**FIGURE 1:**
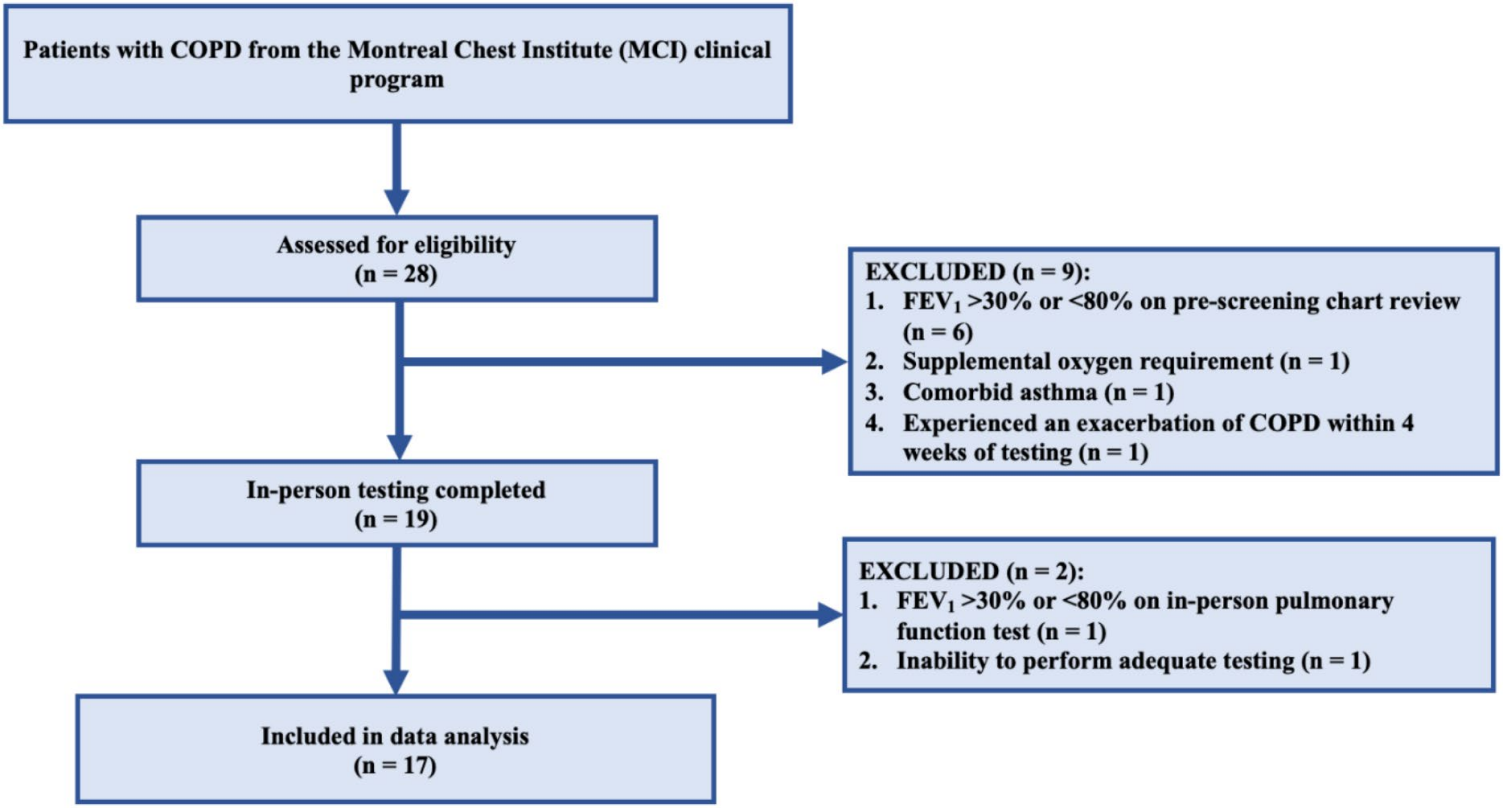
Flow diagram of study participants. COPD, chronic obstructive pulmonary disease; FEV_1_, forced expiratory volume in the first second.

Of the 19 participants who were initially recruited into the study and who completed the in‐person testing visit, the data from a total of 17 participants were included in the final analysis (see Figure 1). Nine participants had GOLD 1 (mild) COPD, and eight participants had GOLD 4 (very severe) COPD. Baseline demographics for all participants and by group are presented in Table 1. The GOLD 1 group was older than the GOLD 4 group and had a higher BMI. Groups were similar in sex and gender representation and reported a similar cumulative cigarette smoking history. Full pulmonary function results by group are presented in Table 2. The mean severity of fixed airflow obstruction (as determined by FEV_1_ percent predicted) was above 90% of predicted in the GOLD 1 group and was below 25% of predicted in the GOLD 4 group. Hyperinflation, gas trapping, and disturbances in lung diffusing capacity were all more severe in the GOLD 4 group.

**TABLE 1:** Participant baseline demographic information.

	All participants	GOLD 1	GOLD 4
Participants, No.	17	9	8
Age, mean: Years (SD)	66.41 (8.31)	71.78 (5.76)	60.38 (6.44)
BMI, mean: Kg/m^2^ (SD)	23.97 (5.05)	26.66 (2.66)	20.98 (5.55)
Smoking History, mean: Pack‐Years (SD)	31.00 (18.44)	29.44 (18.78)	32.75 (19.17)
Female Sex, No. (%)	9 (52.94)	5 (55.56)	4 (50.00)
Male Sex, No. (%)	8 (47.06)	4 (44.44)	4 (50.00)

Abbreviations: %, percent of participants in group; BMI, body mass index; GOLD, global initiative for chronic obstructive lung disease classification; Kg, kilograms; m, meter; No., number.

**TABLE 2:** Full pulmonary function of GOLD 1 and GOLD 4 group participants.

	GOLD 1		GOLD 4	
	Valid	Value	Valid	Value
Post‐bronchodilator spirometry				
FEV_1_, mean: Liters (mean % predicted)	9	2.39 (91.44)	8	0.68 (24.25)
FVC, mean: Liters (mean % predicted)	9	3.91 (114.56)	8	2.39 (66.38)
FEV_1_/FVC ratio, mean	9	0.61	8	0.29
Lung volumes (Plethysmography)				
RV, mean: Liters (mean % predicted)	9	2.69 (119.89)	8	4.92 (247.75)
TLC, mean: Liters (mean % predicted)	9	6.06 (103.44)	8	7.02 (125.88)
RV/TLC ratio, mean (mean % Predicted)	9	43.67 (112.1)	8	70.25 (199.63)
Diffusion capacity				
DLco, mean: mL/min/mmHg (mean % Predicted)	9	13.88 (63.44)	8	8.58 (35.17)

Abbreviations: DLco, diffusing capacity of the lungs for carbon monoxide; FEV_1_, forced expiratory volume in the first second of expiration; FVC, forced vital capacity; GOLD, global initiative for chronic obstructive lung disease classification; min, minute; mL, milliliters; mm Hg, millimeters of mercury; RV, residual volume; TLC, total lung capacity.

**FIGURE 2:**
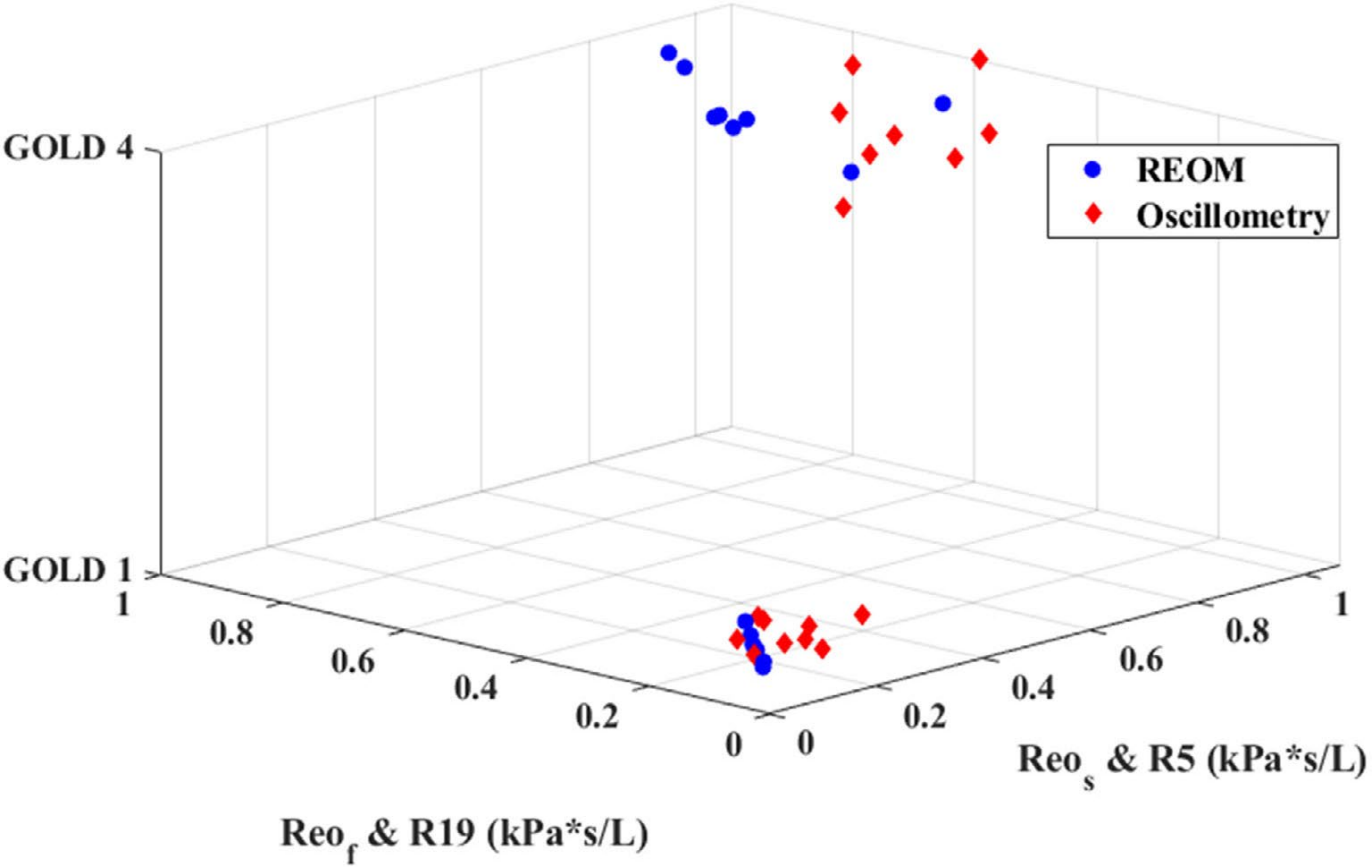
Three‐dimensional plot of the resistance measurements obtained from the conventional spectral oscillometer and from the REOM device for the GOLD 1 (mild) and GOLD 4 (very severe) COPD groups. kPa, kilopascal; L, liters; R19, resistance measured at 19 Hz; R5, resistance measured at 5 Hz; Reo‐f, “fast” resistance during expiration; Reo‐s, “slow” resistance during expiration; s, seconds.

**FIGURE 3:**
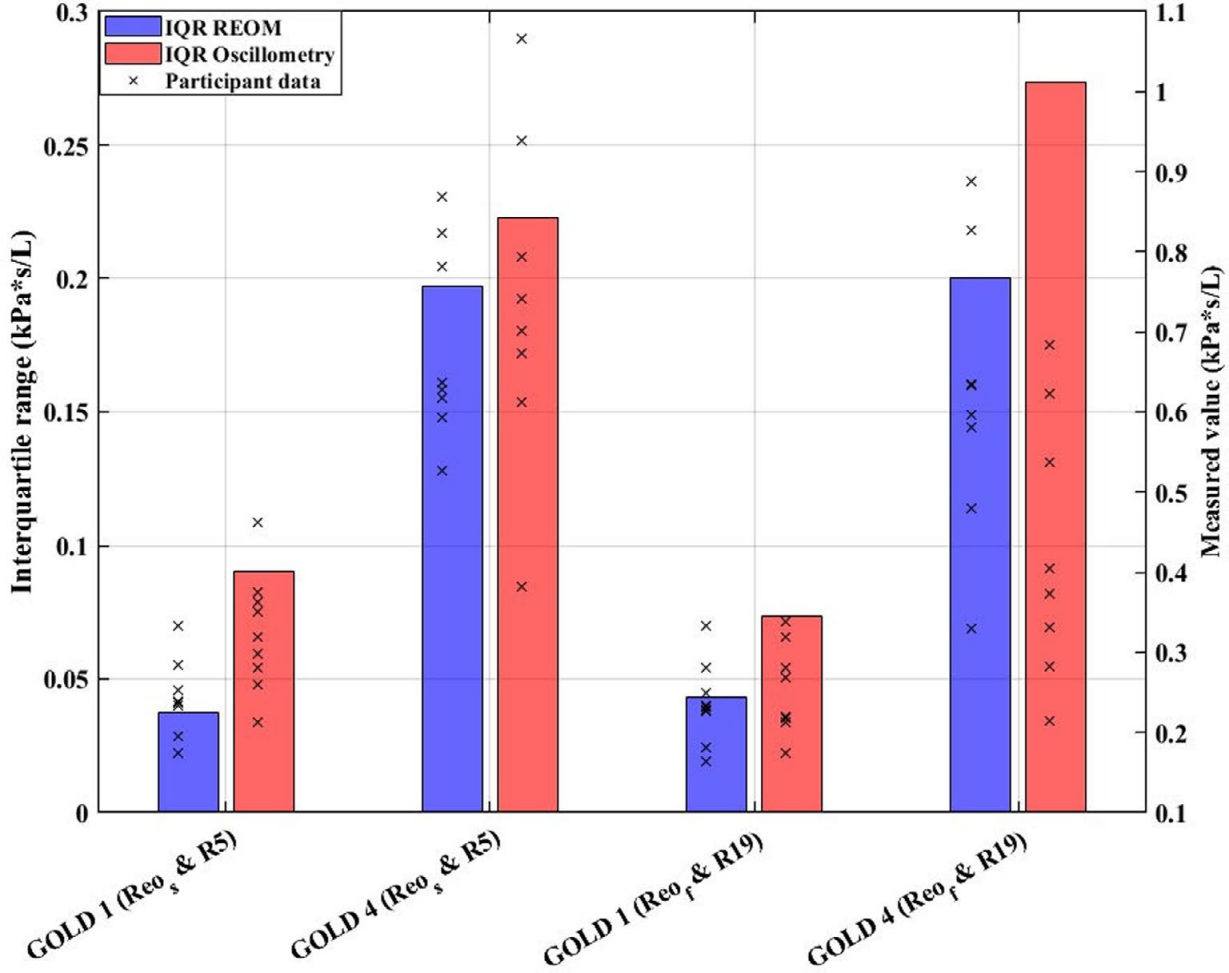
Bar chart of the variation (interquartile range) of resistance measurements obtained by conventional spectral oscillometry and by the REOM device. Results are presented by group. kPa, kilopascal; L, liters; R_19_, resistance measured at 19 Hz; R_5_, resistance measured at 5 Hz; R_eo‐f_, “fast” resistance during expiration; R_eo‐s_, “slow” resistance during expiration; s, seconds.

The main resistance measurements obtained by standard spectral oscillometry and by the REOM device for each group are presented in Table 3 and are graphically presented in Figure 2. The difference between R_5_ and R_19_ (in kPa*s/L) was proportionally greater than the difference between R_eo‐s_ and R_eo‐f_ (in kPa*s/L), though R_5_ measurements were always greater in magnitude than R_19_ measurements and likewise R_eo‐s_ measurements were always greater in magnitude than R_eo‐f_ measurements across both groups. The variation (observed interquartile range) of the REOM‐obtained and oscillometry‐obtained resistance measurements is presented by group in Figure 3.

**TABLE 3:** Resistance as measured by standard spectral oscillometry and by the REOM device.

	GOLD 1		GOLD 4		Wilcoxon rank sum test
	*n*	Value	*n*	Value	
Conventional spectral oscillometry					
R_5_, median: kPa*s/L (IQR)	9	0.319 (0.090)	8	0.721 (0.223)	<0.001
R_19_, median: kPa*s/L (IQR)	9	0.253 (0.071)	8	0.392 (0.252)	<0.001
REOM device					
R_eo‐s_, median: kPa*s/L (IQR)	9	0.237 (0.037)	8	0.632 (0.123)	<0.001
R_eo‐f_, median: kPa*s/L (IQR)	9	0.231 (0.043)	8	0.615 (0.178)	<0.001

Abbreviations: GOLD, global initiative for chronic obstructive lung disease classification; IQR, interquartile range; kPa, kilopascal; L, liters; *n*, number of participants; R_19_, resistance measured at 19 Hz; R_5_, resistance measured at 5 Hz; R_eo‐f_, “fast” resistance during expiration; REOM, rapid expiratory occlusion monitor; R_eo‐s_, “slow” resistance during expiration; s, seconds.

**FIGURE 4:**
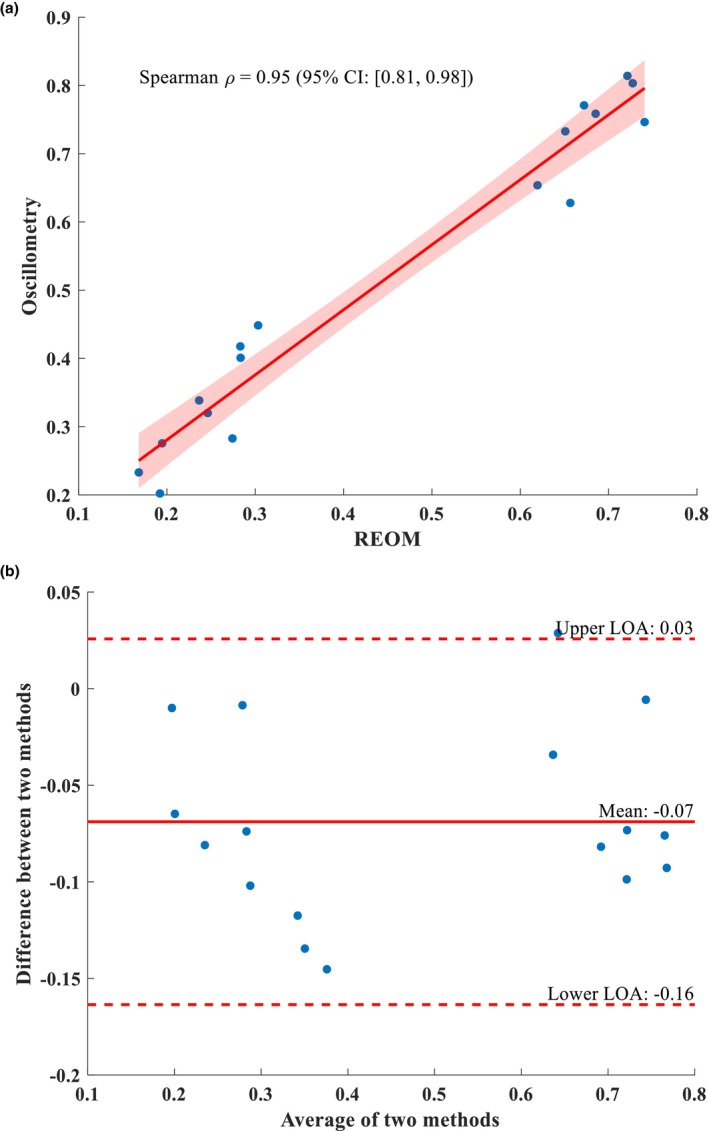
Adjusted Spearman correlation plot (a) and Bland–Altman plot (b) between conventional spectral oscillometry‐obtained R_5_ and REOM‐obtained R_eo‐s_. CI, confidence interval; LOA, limit of agreement; REOM, rapid expiratory occlusion monitor; ρ, rho.

**FIGURE 5:**
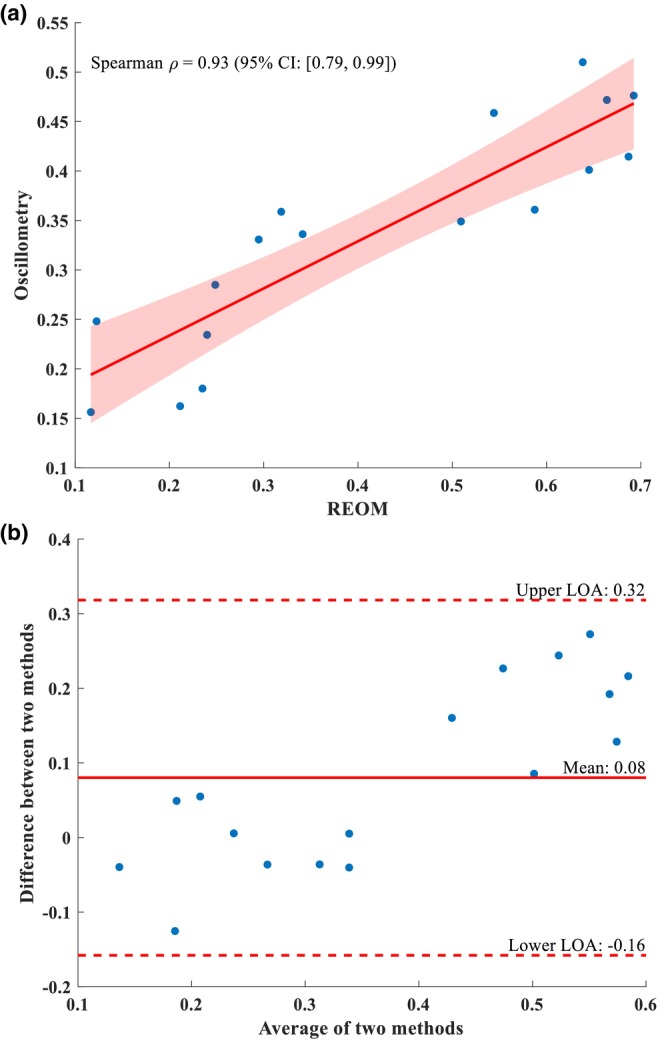
Adjusted Spearman correlation plot (a) and Bland–Altman plot (b) between conventional spectral oscillometry‐obtained R_19_ and REOM‐obtained R_eo‐f_. CI, confidence interval; LOA, limit of agreement; REOM, rapid expiratory occlusion monitor; ρ, rho.

Adjusted correlation and agreement between R_5_ and R_eo‐s_ and between R_19_ and R_eo‐f_, the co‐primary outcomes, are presented in Figures 4 and 5, respectively. Unadjusted analyses are presented in Table S2. The Spearman correlation coefficient for the association between R_5_ and R_eo‐s_ was 0.95 [0.81, 0.98] (*p* < 0.001; see Figure 4a). Bland–Altman analysis yielded a mean difference of −0.07, with upper and lower limit agreement of 0.03 and −0.16, respectively (see Figure 4b). The Spearman correlation coefficient for the association between R_19_ and R_eo‐f_ was 0.93 [0.79, 0.99] (*p* < 0.001; see Figure 5a). Bland–Altman analysis yielded a mean difference of 0.08, with upper and lower limit agreement of 0.32 and −0.16, respectively (see Figure 5b).

**FIGURE 6:**
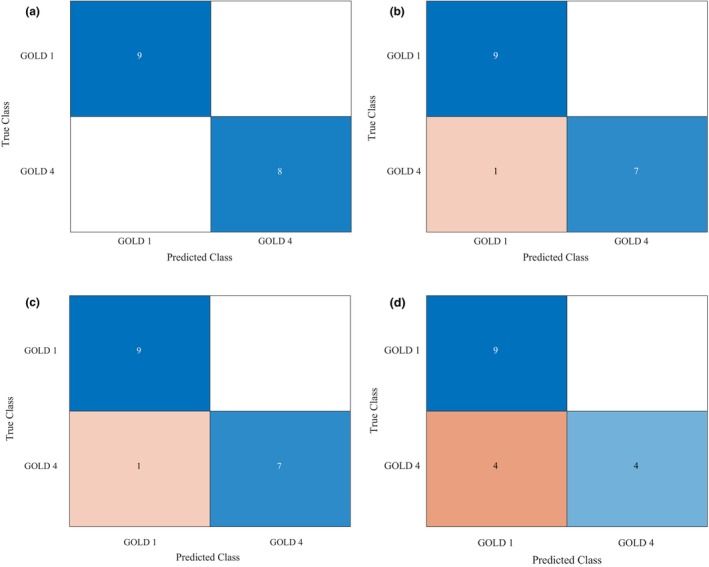
Confusion matrices for the support vector machine (SVM) classification using R_eo‐s_ (a), R_5_ (b), R_eo‐f_ (c), and R_19_ (d) to discriminate between GOLD 1 COPD and GOLD 4 COPD. GOLD, global initiative for chronic obstructive lung disease classification.

Pertaining to all four measurements of resistance (R_eo‐s_, R_eo‐f_, R_5_, and R_19_), the results obtained in participants with GOLD 4 (very severe) COPD were, as expected, significantly higher than the results obtained in GOLD 1 participants. Wilcoxon rank sum tests were statistically significant (*p* < 0.001) for all four parameters (see Table 3), supportive of discriminative capacity. The capacity of resistance measurements to discriminate (classify) COPD severity as evaluated by the SVM technique is presented as confusion matrices in Figure 6. Perfect discrimination was demonstrated by R_eo‐s_. All participants but one were correctly classified by R_5_ and by R_eo‐f_. Finally, four out of 17 participants were incorrectly classified by R_19_. Across all resistance measurements, misclassifications only occurred for participants with GOLD 4 COPD.

User experience (SUS) scores for the REOM device was 98.7 ± 3.3/100 in those with GOLD 1 COPD and 93.1 ± 7.4/100 in those with GOLD 4 COPD, yielding an average score of 96.1 ± 6.1/100 in all participants. Similarly positive results were observed in the numerical section (Questions 1–3) of the PSS in those with GOLD 1 COPD (14.8 ± 0.4/15), those with GOLD 4 COPD (14.0 ± 1.4/15), and in all participants (14.4 ± 1.1/15). In open‐ended PSS responses, participants indicated an appreciation for the lightweight design and ease of use of the REOM device and endorsed a preference for this technique over spirometry/PFT. When asked what they liked “least” about the REOM device, 15 out of 17 participants answered “nothing” or an equivalent statement.

The results from the REOM device using the one‐handed and two‐handed cheek hold methods are provided in Table S3 (no‐cheek hold method results are presented in Table 3). Overall, Friedman test analyses did not reveal any significant intergroup differences across the three techniques. Adjusted and unadjusted Spearman correlation and Bland–Altman analysis for tests conducted while holding the cheeks with one hand or with both hands are both presented in Table S4, yielding similar results to the results obtained with no cheek hold performed.

## 4 DISCUSSION

The present study aimed to determine the concordance and agreement between the two resistance parameters obtained from a handheld lightweight portable device, R_eo‐s_ and R_eo‐f_, with two conventional resistance parameters measured with the standard spectral oscillometer, R_5_ and R_19_, in the adult COPD patient population with mild and very severe fixed airflow obstruction as determined by post‐bronchodilator spirometry. R_eo‐s_ and R_5_ demonstrated the strongest correlation and agreement, while the correlation and agreement between R_eo‐f_ and R_19_ were also excellent, in keeping with prior results in children with asthma (Lundblad, Blouin, et al., 2021). While all four measures of resistance were found to discriminate between mild and very severe COPD, R_eo‐s_ was found to be the single most reliable indicator for the correct classification of disease severity. Participants reported an excellent COPD patient user experience with the REOM device and reported a preference for its use over conventional lung function testing. Three different techniques (associated with varying levels of splinting of the cheeks) during REOM testing were performed in an exploratory manner, and each of the techniques did not yield any significant differences to one another in the results obtained. Furthermore, the method using no cheek hold yielded the closest concordance and agreement with standard spectral oscillometry. Finally, REOM‐obtained measurements generally appeared to be associated with lower variation compared to those obtained through oscillometry. Cumulatively, these results lend support to the potential utility of the handheld portable REOM device as a complementary lung function/monitoring modality in the adult COPD patient population.

Oscillometry is increasingly recognized for its value and relevance as an alternate and complementary diagnostic modality to spirometry, which has led to more widespread adoption and frontline clinical use (Bates et al., 2011; King et al., 2020). In essence, pressure or flow signal, or sound waves of varying frequencies, typically between 5 and 37 Hz, are applied at the mouthpiece to measure mechanical properties of the patient's respiratory system during tidal breathing. As the procedure does not require forced, coordinated, or uncomfortable breathing maneuvers (Bates et al., 2011; King et al., 2020), oscillometry can measure clinically useful parameters of the respiratory system in a more “natural” state (Donohue & Kaminsky, 2024; Lundblad, Siddiqui, et al., 2021). The mechanical impedance (Z_rs_) of the respiratory system is estimated by measuring the forces opposing the oscillating flow signal transmitted through the airways, namely resistance (R_rs_) and reactance (X_rs_) (Kaminsky et al., 2022). Historically, the frequency dependence of resistance, that is, a drop in resistance as oscillation frequency increases, has been considered a hallmark of early obstructive lung disease (Grimby et al., 1968; Michaelson et al., 1975). R_5_ and R_19_ have been considered to correlate with total and large (proximal) airway resistance, respectively (Bickel et al., 2014; Liang et al., 2022). Notably, oscillometry has recently been highlighted as a diagnostic test in the COPD patient population (with particular utility in elderly patients and in those with advanced disease) by the European Respiratory Society (ERS)/American Thoracic Society (ATS) Lung Function Test guidelines (King et al., 2020; Stanojevic et al., 2022). Despite the many advantages of oscillometry, barriers to widespread clinical adoption that are common to spirometry and oscillometry include portability and ease of test result interpretation.

The unmet need to develop a lightweight handheld portable lung function device that offers results concordant with conventional oscillometry is easy to use and interpret, is cost‐effective, and requires no additional in‐person technician or personnel for testing (Kaminsky et al., 2022; Lundblad, Blouin, et al., 2021) may have been effectively addressed through harnessing a modification of the flow‐interrupter technique (Bates et al., 1988; Kaminsky, 2012; Mead & Whittenberger, 1954; Otis & Proctor, 1948). The REOM handheld portable device, previously favorably tested in the pediatric asthma population (Lundblad, Blouin, et al., 2021), enables the measurement of variations in airway pressure and then flow through a brief occlusion of expiration during quiet tidal breathing and thus allows calculation of resistance (Kaminsky, 2012; Otis & Proctor, 1948). In a manner similar to conventional oscillometry, the REOM method removes the need for demanding and uncomfortable forced maximal expiratory maneuvers (Lundblad, Blouin, et al., 2021). During inhalation, a flexible check valve remains open to allow normal airflow. At the very start of expiration, the check valve closes, which momentarily occludes flow, allowing for the generation of a very low pressure (400 Pa). Once the 400 Pa pressure is sensed by the device, the occluding shutter valve opens, allowing expiration to proceed freely. This very brief closure/reopening permits measurements of peak pressure followed by flow after shutter release to measure respiratory system resistance both immediately after opening and again over the subsequent period of early expiration.

Resistance measurements in participants with mild COPD as obtained by both the REOM device and by standard spectral oscillometry were significantly lower than those with very severe COPD. These findings were expected, are in keeping with the nature of the disease, and align with previous literature on oscillometry measurements in the COPD patient population (Crim et al., 2011; Global initiative for chronic obstructive lung disease, 2024; Liang et al., 2022; Liu et al., 2017). While all four resistance measurements were minimally dispersed in the GOLD 1 group, in the GOLD 4 group the parameters (R_19_ in particular) were more dispersed in distribution. Indeed, R_eo‐s_ and low‐frequency oscillometry (i.e., R_5_) are likely best suited to detect the pathology associated with increased resistance at the level of the small peripheral airways in the COPD patient population given the natural history and pathogenesis of the disease. Strong concordance and agreement were found between REOM‐obtained and conventional spectral oscillometry‐obtained resistance measurements. While the correlation between R_eo‐f_ and R_19_ was also strong, the strongest correlation was observed between R_eo‐s_ and R_5_. The lower mean difference and agreement range demonstrated that R_eo‐s_ and R_5_ may be more comparable with one another than R_eo‐f_ and R_19_, observations fully consistent with the only previously published study on the REOM device in obstructive airways disease (Lundblad, Blouin, et al., 2021).

Both the REOM device and standard spectral oscillometry demonstrated a capacity to discriminate between mild and very severe COPD through the measurement of respiratory resistance. Surprisingly, the REOM‐obtained resistance measurements, and R_eo‐s_ in particular, demonstrated a better performance than oscillometry. R_eo‐f_ and R_5_ also demonstrated good discriminatory ability, as did R_19_ to a lesser extent, again in keeping with the literature demonstrating the excellent capacity of R_5_ specifically in detecting COPD (Liang et al., 2022). All misclassifications involving the resistance parameters, particularly pronounced when using R_19_, were observed in the GOLD 4 COPD group. Given that R_5_ has been shown to correlate more closely with FEV_1_ than R_19_ in patients with COPD (Kolsum et al., 2009), and given the characteristic feature of small airway disease in COPD, this again demonstrates the proportionally greater utility of low‐frequency compared with high‐frequency oscillometry (Bickel et al., 2014; Bokov et al., 2021; Handa et al., 2014) and of their analogous measurements using the REOM device in the COPD patient population (Lundblad, Siddiqui, et al., 2021).

Participant feedback through end‐of‐study surveys and questionnaires confirms the favorable user experience with the REOM device. Beyond very high numeric scores, open‐ended responses highlighted mainly positive feedback regarding the size, weight, and ease of use of the REOM device when compared to more established lung function testing methods, even when the device was operated independently by the participant without assistance from the technician. REOM device favorability was demonstrated at both ends of the disease spectrum, supporting the potential for real‐world use in the outpatient setting.

Patients with COPD, particularly those who are older adults and those with advanced disease, are highly vulnerable and are frequent users of health system resources including emergency department visits as well as recurrent hospitalization. Because acute exacerbations of COPD are a leading cause of hospitalization among all adult chronic diseases, innovations in chronic disease management including remote patient monitoring solutions represents an important and growing field of clinical research (Coutu et al., 2023). The results of the present study, that a lightweight portable handheld device can be used to obtain correlates of respiratory system resistance, and with a favorable COPD patient user experience, are highly complementary to recent work establishing the feasibility of home autonomous use of remote patient monitoring technologies such as biometric wearable devices in patients with COPD (Iorio et al., 2024) as well as the demonstration of the high value of detailed pathophysiological data collected during acute exacerbations of COPD from the home environment (Coutu et al., 2024). Future applications of the REOM device include lung function monitoring in community practice, in remote or rural settings, as well as in remote patient monitoring in the event of a future pandemic scenario, particularly for vulnerable patients (older adults and patients with very severe disease). The equivalence of the autonomous measurement method, that is, without any cheek hold, to assisted measurement with two‐handed cheek holding techniques lends further support to the feasibility of autonomous home use. The frequency dependence of resistance first described by Grimby et al. (1968) and whose measurement was simplified by Michaelson et al. (1975) is now considered a hallmark of small airway disease (Kaminsky et al., 2022; Mead & Whittenberger, 1954). Because frequency dependence of resistance is also observed in obesity and upper airway shunt (Albuquerque et al., 2015; Bates et al., 2011; Cauberghs & Van de Woestijne, 1989; Watson & Pride, 2005), cheek hold is recommended when performing oscillometry to attenuate the loss of the forcing signal in the compliant tissues of the cheeks, mouth floor, soft palate, and pharynx (Bates et al., 2011; Cauberghs & Van de Woestijne, 1989; King et al., 2020). The finding of no difference between the various levels of cheek hold, in this context, in the REOM measurements may be in part due to the fact that while with conventional oscillometry the *machine* delivered (“forced”) the test signal down the airways, with REOM, the participant produced the test signal. The low level of pressure used in the REOM measurements may also be contributory, and indeed both REOM and oscillometry are performed during quiet tidal breathing. Overall, we report this lack of shunt effect with caution and highlight the need to confirm this observation in larger studies.

This study has important limitations. The study was conducted at a single site, an academic tertiary center, which may introduce bias and affect external generalizability. The sample size may also limit the findings and affect the strength of the observed correlation between resistance parameters. Only participants with GOLD 1 or 4 COPD were recruited in the study, in part to ensure that the feasibility and ease of use in advanced disease was established as well as to allow for the testing of REOM parameter discriminative capacity between subgroups along the spectrum of the same disease. The results of the present study cannot be extrapolated to moderate and severe COPD, and therefore future investigation in a much larger COPD cohort (with all severity subcategories well‐represented) is required. Finally, in the event of more widespread use of the REOM device, normative values (Berger et al., 2021) and technical standards (King et al., 2020) including optimal technique during testing and the maximum permitted coefficient of variation amongst measurement sets, will need to be developed and disseminated for appropriate and reproducible clinical use.

In summary, the REOM‐obtained measurements of respiratory resistance R_eo‐s_ and R_eo‐f_ are highly concordant with the standard spectral oscillometry‐obtained parameters R_5_ and R_19_, respectively, in a sampling of patients with mild and very severe COPD. The strongest correlation was observed between R_eo‐s_ and R_5_. R_eo‐s_ was particularly strong at classifying COPD disease severity. Cheek‐hold techniques did not significantly affect the values obtained, and COPD user experience was favorable, both supporting the applicability of the device towards remote patient chronic disease monitoring. Further work to confirm these results, to test the device in moderate and severe disease, and to demonstrate the feasibility of home monitoring is needed. Considering the current and ongoing challenges in the diagnosis and management of COPD, REOM device portability, ease of operation and interpretation, and the potential for remote patient monitoring are highly promising features that may be applied towards the effective and efficient clinical management of high‐risk patients, particularly older adults and patients living with advanced COPD.

## AUTHOR CONTRIBUTIONS

BAR conceptualized and designed the methodology of the study, obtained funding, and drafted the study protocol with TM. RJD, LKAL, and SBG reviewed the study protocol. FAC, DM, and OCI managed the project and data collection. FAC, DM, OCI, SN, TM, and BAR curated and analyzed the data and wrote the original draft of the manuscript. All authors read and approved the final version of the manuscript.

## FUNDING INFORMATION

This study was funded by the following grants: Ministère de l'Éducation et le Ministère de l'Enseignement Supérieur Innovation and Office of Innovation & Partnerships (I&P: McGill University & Thorasys Thoracic Medical Systems, Inc.), the McGill University Health Centre (MUHC) Department of Medicine Contract Academic Staff (CAS) Research Award, and the Respiratory Epidemiology and Clinical Research Unit (RECRU) summer studentship Research Award.

## CONFLICT OF INTEREST STATEMENT

FAC, DM, OCI, SN, TM, and SBG report no disclosures or conflicts of interest. LKAL is an employee of Thorasys Thoracic Medical Systems Inc., the manufacturer of the tremoflo C‐100 and worldwide license holder for the REOM device. RJD is a co‐holder of the REOM patent (Spiro‐Tech Medical Inc., 20% shareholder) and has licensed it to Thorasys Thoracic Medical Systems Inc.; has investigator‐initiated grants from AstraZeneca and Boehringer Ingelheim, unrestricted grants from Thorasys; and has received honoraria from the European Respiratory Society (Speaker) and the Association des Pneumologues de la Province du Québec (Speaker). BAR reports the following disclosures/conflicts of interest: Honoraria from the Canadian Thoracic Society (CTS—COPD Educational Event (Speaker) and Content Creator (educational materials)), CHEST (Content Creator (educational materials)), Respiplus non‐profit (Content Creator (educational materials)), Alberta Kinesiology Association (AKA—Content Creator (educational materials)), Association des Pneumologues de la Province du Québec (APPQ—presenter), McGill University Continuing Professional Development (CPD—COPD Educational Event (Speaker)), GSK (Speaker and Moderator), AZ (Speaker and Moderator), and COVIS (COPD Educational Event (Speaker)); Research funding as Principal Investigator from the McGill University Health Centre (MUHC) Department of Medicine Contract Academic Staff (CAS) Research Award, Montreal General Hospital Research Award, Trudell Medical International Unrestricted Investigator‐Initiated Operating Grant, AstraZeneca Unrestricted Investigator‐Initiated Operating Grant, DiaSorin Unrestricted Investigator‐Initiated Operating Grant, the Quebec Respiratory Health Network (QHRN), Ministère de l'Éducation et le Ministère de l'Enseignement Supérieur Innovation and Office of Innovation & Partnerships (I&P: McGill University & Thorasys Thoracic Medical Systems, Inc.), and the MUHC Foundation/MCI Respiratory Research Campaign Innovation Grant; and reception of in‐kind support (placebo and intervention) for research from Amazentis, and reception of in‐kind support (diagnostic device(s)) for research from Thorasys Inc. and Restech. BAR maintained full autonomy regarding the scientific conduct of the study, all scientific decisions, all data analyses, and the decision to publish the study findings.

## ETHICS STATEMENT

The study was approved by the MUHC Research Ethics Board (REB study number: 2023‐9001). All participants provided both written and verbal informed consent prior to participation. This study received approval from Health Canada for Class II Investigational Testing Authorization (ITA: 355823) and was registered on ClinicalTrials.gov (NCT 05913323).

## Supporting information


Tables S1–S4.

## Data Availability

Due to data privacy regulations, individual participant data collected during this study is not publicly accessible. However, access to anonymized data may be granted upon evaluation by the trial management group. Additional documents will also be available upon inquiry. All requests should be directed to the corresponding author (BAR).
