# Supplementary material for: Measuring lung mechanics in patients with COPD using the handheld portable rapid expiratory occlusion monitor (REOM): A cross‐sectional study
Source: Physiol Rep. 2025 Apr 7;13(7):e70307. doi: 10.14814/phy2.70307 (PMC11973731; doi:10.14814/phy2.70307)
Supplement: Supplementary file 1 — Tables S1–S4. [file PHY2-13-e70307-s001.docx]

**Supplementary Material**

Measuring Lung Mechanics in Patients with COPD using the Handheld Portable Rapid Expiratory Occlusion Monitor (REOM): A Cross-Sectional Study

Felix-Antoine Coutu, B.Sc.^1,2^, Dany Malaeb^1^, Olivia C Iorio, B.Sc.^1,2^, Seyedfakhreddin Nabavi, Ph.D.^1^, Tom McFarland, MD^1,2^, Lennart K.A. Lundblad, PhD^3,5^, Ron J. Dandurand^2,3,4,6,7^, MD, Stewart B. Gottfried, MD^2,3,4,7^, Bryan A. Ross, MD^1,2,4,7*^

**Affiliations:**

^1^Respiratory Epidemiology and Clinical Research Unit, Centre for Outcomes Research and Evaluation, Research Institute of the McGill University Health Centre, Montreal, QC, Canada.

^2^Department of Medicine, McGill University, Montreal, QC, Canada.

^3^ Meakins-Christie Laboratories, McGill University, Montreal, Quebec, Canada.

^4^Montreal Chest Institute, McGill University Health Centre, Montreal, QC, Canada.

^5^THORASYS Thoracic Medical Systems Inc., Montreal, Quebec, Canada.

^6^Lakeshore General Hospital, Pointe-Claire, Canada; Ste-Anne Hospital, Ste-Anne-de-Bellevue, Canada

^7^Translation Research in Respiratory Diseases Program, Research Institute of the McGill University Health Centre, McGill University, Montreal, Quebec, Canada

**Correspondence** (*):

Dr. Bryan A. Ross

Respiratory Epidemiology and Clinical Research Unit, Centre for Outcomes Research and Evaluation, Research Institute of the McGill University Health Centre

5252 De Maisonneuve, Suite 3D.57, Montréal, QC H4A 3S5, Canada

E-mail: [bryan.ross@mcgill.ca](mailto:bryan.ross@mcgill.ca).

**Table S1.** Participant Satisfaction Survey

|  | Participant Satisfaction Survey (REOM Device) | Strongly Disagree | Disagree | Neutral | Agree | Strongly Agree |
| --- | --- | --- | --- | --- | --- | --- |
|  |  | 1 | 2 | 3 | 4 | 5 |
| 1 | I liked doing the REOM device test (my reaction to the REOM test is positive). |  |  |  |  |  |
| 2 | It was easy to do the rest with the REOM device. |  |  |  |  |  |
| 3 | If my doctor asked, I would agree to use the REOM device at home. |  |  |  |  |  |
| 4 | What I liked best about using the REOM device (open-ended). |  | | | | |
| 5 | What I liked least about using the REOM device (open-ended). |  | | | | |

Abbreviation: REOM: rapid expiratory occlusion monitor.

**Table S2.** Unadjusted Correlation and Agreement for R_5_ & R_eo-s_ and for R_19_ & R_eo-f_ for the No Cheek Hold Technique

|  | **Spearman correlation test** | | | **Bland-Altman test** | | |
| --- | --- | --- | --- | --- | --- | --- |
|  | Correlation coefficient | 95% interval | P value | Mean difference | Upper limit agreement | Lower limit agreement |
| R_5_ & R_eo-s_ (No Cheek Hold) | 0.87 | [0.64, 0.95] | <0.001 | -0.07 | 0.15 | -0.28 |
| R_19_ & R_eo-f_ (No Cheek Hold) | 0.76 | [0.38, 0.93] | <0.001 | 0.08 | 0.34 | -0.18 |

Abbreviations: R_eo-f_: ‘fast’ resistance during expiration; R_eo-s_: ‘slow’ resistance during expiration; R_5_: Resistance measured at 5Hz; R_19_: Resistance measured at 19 Hz.

**Table S3.** Resistance Measurements Obtained from the REOM Device Using One-Handed and Two-Handed Cheek Hold Techniques

|  | **GOLD 1** | | **GOLD 4** | |
| --- | --- | --- | --- | --- |
|  | n | Value | n | Value |
| **REOM (One-Handed Cheek Hold)** | | | | |
| R_eo-s_, median: kPa*s/L (IQR) | 8 | 0.257 (0.089) | 8 | 0.697 (0.152) |
| R_eo-f_, median: kPa*s/L (IQR) | 8 | 0.254 (0.088) | 8 | 0.708 (0.198) |
| **REOM (Two-Handed Cheek Hold)** | | | | |
| R_eo-s_, median: kPa*s/L (IQR) | 9 | 0.283 (0.066) | 7 | 0.698 (0.101) |
| R_eo-f_, median: kPa*s/L (IQR) | 9 | 0.282 (0.068) | 7 | 0.703 (0.094) |

Abbreviations: GOLD: global initiative for chronic obstructive lung disease classification; kPa: kilopascal; L: Liters; n: number of participants; REOM: rapid expiratory occlusion monitor; R_eo-f_: ‘fast’ resistance during expiration; R_eo-s_: ‘slow’ resistance during expiration; R_5_: Resistance measured at 5 Hz; R_19_: Resistance measured at 19 Hz; s: seconds; IQR: interquartile range.

**Table S4.** Adjusted and Unadjusted Correlation and Agreement for R_5_ & R_eo-s_ and for R_19_ & R_eo-f_ Using One-Handed and Two-Handed Cheek Hold Techniques

|  | **Spearman Correlation Test** | | | **Bland-Altman Test** | | |
| --- | --- | --- | --- | --- | --- | --- |
|  | Correlation coefficient | 95% interval | P value | Mean difference | Upper limit agreement | Lower limit agreement |
| Adjusted | | | | | | |
| R_5_ & R_eo-s_ (One-Handed Cheek Hold) | 0.96 | [0.82, 1] | <0.001 | -0.03 | 0.07 | -0.14 |
| R_19_ & R_eo-f_ (One-Handed Cheek Hold) | 0.86 | [0.63, 0.96] | <0.001 | 0.12 | 0.41 | -0.16 |
| R_5_ & R_eo-s_ (Two-Handed Cheek Hold) | 0.89 | [0.66, 0.98] | <0.001 | -0.04 | 0.08 | -0.16 |
| R_19_ & R_eo-f_ (Two-Handed Cheek Hold) | 0.84 | [0.62, 0.93] | <0.001 | 0.13 | 0.41 | -0.15 |
| Unadjusted | | | | | | |
| R_5_ & R_eo-s_ (One-Handed Cheek Hold) | 0.94 | [0.80, 0.99] | <0.001 | -0.03 | 0.17 | -0.24 |
| R_19_ & R_eo-f_ (One-Handed Cheek Hold) | 0.63 | [0.21, 0.84] | 0.011 | 0.12 | 0.50 | -0.26 |
| R_5_ & R_eo-s_ (Two-Handed Cheek Hold) | 0.89 | [0.67, 0.97] | <0.001 | -0.04 | 0.22 | -0.30 |
| R_19_ & R_eo-f_ (Two-Handed Cheek Hold) | 0.66 | [0.18, 0.92] | 0.007 | 0.13 | 0.47 | -0.20 |

Abbreviations: R_eo-f_: ‘fast’ resistance during expiration; R_eo-s_: ‘slow’ resistance during expiration; R_5_: Resistance measured at 5 Hz; R_19_: Resistance measured at 19 Hz.
